# Salt Acclimation of Cyanobacteria and Their Application in Biotechnology

**DOI:** 10.3390/life5010025

**Published:** 2014-12-29

**Authors:** Nadin Pade, Martin Hagemann

**Affiliations:** Institut für Biowissenschaften, Abteilung Pflanzenphysiologie, Universität Rostock, Albert-Einstein-Str. 3, D-18059 Rostock, Germany; E-Mail: nadin.pade@uni-rostock.de

**Keywords:** compatible solute, genome mining, ion export, regulation, salt sensing

## Abstract

The long evolutionary history and photo-autotrophic lifestyle of cyanobacteria has allowed them to colonize almost all photic habitats on Earth, including environments with high or fluctuating salinity. Their basal salt acclimation strategy includes two principal reactions, the active export of ions and the accumulation of compatible solutes. Cyanobacterial salt acclimation has been characterized in much detail using selected model cyanobacteria, but their salt sensing and regulatory mechanisms are less well understood. Here, we briefly review recent advances in the identification of salt acclimation processes and the essential genes/proteins involved in acclimation to high salt. This knowledge is of increasing importance because the necessary mass cultivation of cyanobacteria for future use in biotechnology will be performed in sea water. In addition, cyanobacterial salt resistance genes also can be applied to improve the salt tolerance of salt sensitive organisms, such as crop plants.

## 1. Introduction

Cyanobacteria are an ancient monophyletic group of the domain bacteria that have colonized all light-exposed habitats on the Earth. During their long evolution, cyanobacteria have developed mechanisms to adapt to a broad range of environmental factors [1]. Salinity is an important abiotic stress for cyanobacteria in aquatic and terrestrial ecosystems. The term salinity refers to the total concentration of dissolved inorganic ions. Thus, salinity can not only vary in total value but also in composition. It is known that specific inorganic ions exert direct toxic effects on cyanobacteria, which can restrict the occurrence of sensitive strains to certain environments [2]. However, in this review, we will concentrate on acclimation to high and fluctuating salinity in different amounts of NaCl or seawater, which contains NaCl as the predominant salt. The amount of salt is inversely correlated with the amount of water in a solution, so increased salinity reduces water availability and simultaneously induces osmotic stress. Generally, unicellular cells such as cyanobacteria maintain a constant cellular ionic and osmotic composition to sustain water uptake via osmosis and to create the turgor pressure that is necessary for cell enlargement and growth, which is hyperosmotic compared to the surrounding medium. Hence, a change in the external salt concentration and/or water availability poses a challenge for the cellular metabolism and for cell survival. A quick response to fluctuations in salt and/or osmotic concentration is important for survival in many habitats.

Two crucial problems occur in the presence of high salt concentrations: water availability is lowered, and the concentration of ions is increased. Large concentrations of inorganic ions in the cytoplasm can have toxic effects on cellular metabolism. To ensure water uptake via osmosis, the cytoplasmic concentration of osmotically active compounds must be higher than that of the surrounding medium [3]. Microorganisms have developed two effective strategies for the osmotic acclimation of the cytoplasm to changing salt concentrations: the “salt-in-strategy” and the “salt-out-strategy” [4,5]. Organisms that use the “salt-in-strategy” accumulate very large numbers of inorganic ions (2–3 M, mainly KCl) in the cytoplasm to ensure water uptake and turgor pressure. However, this energetically cheap strategy is restricted to a small number of true halophiles, e.g., aerobic halophilic archaea of the order *Halobacteriales* [4], the anaerobic, halophilic bacteria of the order *Halanaerobiales* [6] or the extreme halophilic organism *Salinibacter ruber* [7]. All of these prokaryotes are highly specialized and able to propagate in nearly saturated brine. During evolution, the entire metabolism of these organisms has become adapted to such high salt concentrations, *i.e.*, all proteins have developed special structures to remain active in a salt-rich medium [8,9]. Most prokaryotes including cyanobacteria and all eukaryotic microorganisms use the “salt-out-strategy” to acclimate to high or changing salt concentrations. The basis of this strategy is to keep the internal ion concentration low, and the necessary osmotic acclimation is achieved via the accumulation of small organic molecules called compatible solutes. This strategy has two parts. First, the cells accumulate compatible solutes that do not interfere with metabolism at high concentrations [10], and second, they actively export inorganic ions that steadily diffuse along their electrochemical gradients into the cytoplasm.

### Goal of the Review

The understanding of the complex physiology and molecular biology of cyanobacterial acclimation to salt stress becomes increasingly important because cyanobacteria will be used for the biotechnological production of fuels and chemical feedstocks in the future. The necessary mass cultivation must be performed in sea water to conserve freshwater resources and to minimize the growth of competing organisms. However, salt acclimation itself is a highly energy demanding process and might interfere with this future application of cyanobacteria. Thus, the engineering of cyanobacterial producer strains must encompass the selection or the generation of an efficient salt acclimation strategy. There have been many recent reviews on bacterial [11,12,13,14] and cyanobacterial [2,5,15,16,17,18] salt stress acclimation. In this review we will briefly explain the basal salt acclimation strategy of cyanobacteria that was elucidated using model cyanobacteria with different degrees of salt tolerance. While this process is quite well understood, the sensing of salt stress and the regulation of the complex cellular reorganization is much less well understood. Hence, we will identify new insights in the regulation of salt acclimation in bacteria and cyanobacteria and will mention open questions that deserve future attention. Finally, emerging aspects of salt acclimation related to cyanobacterial biotechnology will be described.

## 2. Compatible Solutes–Universal & Useful

The concept of the compatible solute was introduced by Brown [10]. Compatible solutes are organic molecules with low molecular masses that usually lack a net charge. They can be accumulated to high (molar) concentrations in the cytoplasm without interfering with the cellular metabolism. The main function of compatible solutes is to increase the internal osmolality, which ensures water uptake and establishes turgor pressure. The water exclusion model is currently the best hypothesis explaining the molecular action of compatible solutes [11,19,20]. According to this model, compatible solutes do not directly interfere with macromolecules such as proteins and/or membranes; rather, they change the water structure. The remaining free water is then directed into the vicinity of the macromolecular surface so that the hydration shell is retained and denaturation is prevented.

A great chemical variety of compatible compounds exists, ranging from amino acids and their derivatives to sugars and polyols [4,21]. Interestingly, in cyanobacteria, the chemical structure of the major compatible solute correlates with the final salt tolerance limit. Freshwater strains (resisting up to 600 mM NaCl equivalent to full seawater conditions) accumulate the sugars trehalose and/or sucrose. These compounds are also effective protectors against desiccation [15]. The moderately halotolerant strains (which grow in freshwater up to a salt level three times that of seawater) such as the model organism *Synechocystis* sp. PCC 6803 characteristically accumulate glucosylglycerol (GG) as the major compatible solute. Halophilic cyanobacteria that can tolerate salt concentrations up to saturation synthesize the compatible solute glycine betaine [5,22]. In addition to these principal groups of cyanobacterial compatible solutes, some compounds are restricted to certain strains or are only accumulated in minor amounts. For example, some hypersaline strains have been reported to accumulate glutamate betaine as a second compatible solute in addition to glycine betaine [23]. A potential role for proline as a compatible solute has been proposed [24], because proline over-accumulation confers enhanced salt tolerance to *Nostoc muscorum*. As a minor compatible solute, proline has also been found transiently in salt-shocked cyanobacteria, e.g., in cells of the hypersaline strain *Synechococcus* sp. PCC 7418 [25]. Recently, the occurrence of glucosylglycerate (GGA) has been reported as a secondary compatible solute in marine cyanobacteria, such as strains of the picoplanktonic *Prochlorococcus/Synechococcus* clade [26]. GGA is a rather uncommon compatible solute because it is negatively charged. This charge seems to function to balance the positive charge of K^+^, an ion accumulated in salt-stressed cells, because the internal pool of glutamate that fulfills this function in other cyanobacteria is limited in the N-poor environment of the central ocean.

Recently, the genes coding for the enzymes that synthesize these compatible solutes (discussed below) have been identified and could be used to search the more than 120 existing cyanobacterial genome sequences [27] for the capacity to synthesize compatible solutes and identify potential salt tolerant strains. Such searches have revealed deviations from the expected pattern [17]. For example, the genome of *Crocosphaera watsonii* strain WH8501, a marine N_2_-fixing unicellular cyanobacterium [28], did not contain the expected genes for the synthesis of GG. Instead, we found a bacterial-like gene for the synthesis of trehalose that was accumulated as the only compatible solute in the cytoplasm of this marine cyanobacterium [29]. Additionally, the genomes of the marine *Prochlorococcus* strains lack genes for GG synthesis. These strains accumulate sucrose as their main compatible solute and often accumulate GGA as a minor component [26]. The marine filamentous N_2_-fixing cyanobacterium *Trichodesmium erythraeum* IMS101 plays an important role as an N-fertilizer in the oceans [30] and represents an interesting example, because no genes that are known to code for enzymes involved in the biosynthesis of any compatible solute from prokaryotes, eukaryotes or archaea were found in the genome of *Trichodesmium*. Indications also exist that oligosaccharides might play a role as compatible solutes among Nostocales [31,32]. Certainly there are many more compatible compounds synthesized by members of the cyanobacterial radiation that have not been discovered yet. Recently, the spectrum of compatible solutes was investigated in a cyanobacterial community harvested from living stromatolites on the coast of Australia. To the surprise of the authors, GG was not present, but they found indications that glycine betaine and several unknown saccharides were accumulated. Moreover, the compatible solute trimethylamine-*N*-oxide was detected, which had not been previously reported in cyanobacteria [33]. However, these were field samples that also certainly contained many other microorganisms, including various heterotrophic bacteria. Thus, cyanobacterial synthesis of trimethylamine-*N*-oxide and other unusual compatible solutes has not yet been verified.

In addition to their role as osmolytes in the cytoplasm of salt-stressed cells, compatible solutes have other valuable functions. It has been shown that these compounds can directly protect enzymes and membranes against denaturation not only due to salt stress but also due to high or low temperatures [34,35]. Thus, these compounds are most likely also protective in living cells, which explains why the accumulation of even small amounts of compatible solutes results in the increased tolerance to multiple environmental stresses [36,37]. Their action as a universal stress protectant is reflected by their designation as low molecular weight chaperones acting together with the protein chaperone family [38]. These features make it highly interesting to use these compounds *in vivo*, e.g., after the expression of certain genes to support cyanobacterial mass cultivation for biotechnology under unfavorable conditions, or *in vitro*, e.g., as supplement to cosmetic and pharmaceutical products [39,40].

### 2.1. Insights Regarding Compatible Solute Synthesis

**Figure 1:**
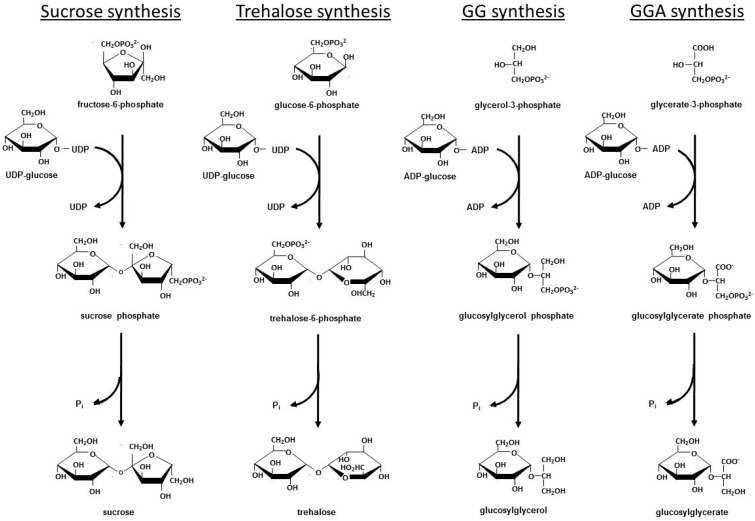
Biochemical pathways for the synthesis of sucrose, trehalose (OtsAB pathway, used only by *Crocosphaera watsonii* in cyanobacteria), glucosylglycerol (GG) and glucosylglycerate (GGA).

As photoautotrophic organisms, cyanobacteria prefer the *de novo* synthesis of compatible solutes. The biosynthetic pathways and the genes coding for the corresponding enzymes are known for all principal compatible solutes in cyanobacteria [5]. Sucrose, GG and GGA are all synthesized by two-step pathways. In the first step, a sugar-phosphate synthase (e.g., GG-phosphate synthase (GGPS)) synthesizes a phosphorylated intermediate by condensing a sugar nucleotide (for sucrose, UDP-glucose is used; for GG and GGA, ADP-glucose is used) and the corresponding phosphorylated sugar molecule (e.g., fructose 6-phosphate, glycerol 3-phosphate, glycerate 3-phosphate). In the second step, the phosphorylated compatible solute is then hydrolyzed to the final product by a specific phosphatase (e.g., GG-phosphate phosphatase (GGPP)) (Figure 1) [26,41,42,43,44]. In enterobacteria and plants, trehalose synthesis also is performed by a two-step mechanism using the so-called OtsAB pathway [11], whereas cyanobacteria usually use the so-called TreY/TreZ pathway for trehalose synthesis. This pathway begins with glycogen as a precursor. In the first reaction the final sugar bond is changed from the α-1,4 to the α-1,1 configuration, and then the two final glucose moieties are cleaved off as trehalose from the high molecular weight precursor, as was first shown in the freshwater model strain *Nostoc* (*Anabaena*) sp. PCC 7120 [45]. Recently, the bacteria-like OtsAB pathway for trehalose synthesis was identified as restricted to the marine cyanobacterium *Crocosphaera.* This strain most likely received the gene via horizontal gene transfer from heterotrophic marine bacteria [29]. An additional trehalose synthesis pathway may exist in cyanobacteria because salt-induced trehalose accumulation was detected in *Microcystis aeruginosa*, however none of the genes for the two known trehalose synthesis pathways is present in its genome (Hagemann and Dittmann, unpublished observation). The cyanobacterial glycine betaine synthesis also differs from the mechanism used by the majority of organisms, which generate glycine betaine by the two-step oxidation of choline [11]. Cyanobacteria and some other phototrophic bacteria instead use glycine as precursor that is methylated in three subsequent reactions, which are performed by two different enzymes, as shown first in the halophilic model cyanobacterium *Aphanothece halophytica* [46].

After the initial description of the enzymes and the identification of the corresponding genes in model cyanobacteria, similar genes and biosynthetic pathways for these compatible solutes have been found in many more strains. For sucrose, at least three different types of sucrose 6-phosphate synthases (SPS) have been identified in various cyanobacteria [17,47]. However, they seem to share a common evolutionary origin and are believed to be the precursors of plant SPS [48]. Sucrose accumulation and its synthesis via SPS and sucrose 6-phosphate phosphatase (SPP) have been recently identified as important factors for the extreme desiccation tolerance of a novel cyanobacterial isolate *Gloeocapsopsis* AAB1 from the Atacama Desert [49]. GG synthesis enzymes have been cloned from strains of the biotechnologically important cyanobacterium *Arthrospira* sp. [50]. As discussed above, this knowledge also allows the prediction of novel producers of glycine betaine form genome sequencing projects [51] and the computational prediction of osmoregulatory networks in cyanobacteria [52].

### 2.2. Compatible Solute Transport

**Figure 2:**
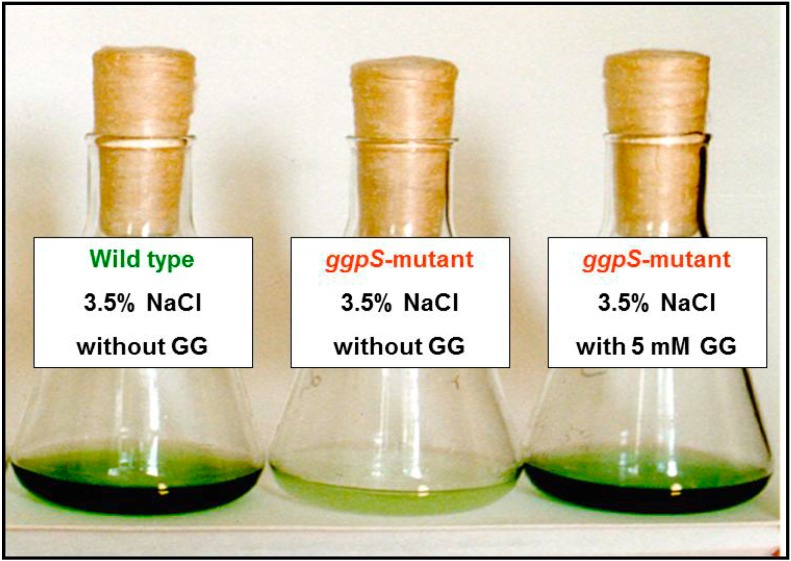
Comparison of growth of the *Synechocystis* sp. PCC 6803 wild type and the mutant defective in the gene for glucosylglycerol (GG) synthesis (*ggpS*) in medium supplemented with 3.5% NaCl. The wild type can grow, whereas the mutant cannot grow at this salinity. Supplementation of the salt medium with 5 mM GG restores the salt tolerance of the mutant, because the compatible solute is taken up by the mutant cells and becomes accumulated to levels comparable to wild-type cells.

Heterotrophic bacteria preferentially accumulate compatible solutes from the environment. Many transport systems have been characterized in a variety of heterotrophic bacteria [12,53,54,55]. The activity and expression of these uptake systems are controlled by the salinity of the external medium [56]. It is also known that *E. coli* and *B. subtilis* can accumulate many more compatible solutes via transport from the environment than they can synthesize *de novo.* Many of the well-characterized transport systems belong to the group of primary active ATP-binding-cassette-(ABC)-type transporters [11]. Despite a preference for *de novo* synthesis of compatible solutes, cyanobacteria also possess transporters for compatible solutes. Initially, an uptake system for glycine betaine was identified, which occurred only in strains able to synthesize this osmolyte *de novo* [57,58]. Subsequently, the existence of an active-transport system for exogenous GG was found in the cyanobacterium *Synechocystis* sp. PCC 6803, which also transports the solutes sucrose and trehalose, albeit with a lower affinity [59,60]. The transporter belongs to the ABC transporter family and consists of four subunits, three of which are coded by genes organized in an operon [61,62]. The identification of these genes allows the generation of mutants to analyze the function of the transporter. Interestingly, a transport mutant of *Synechocystis* sp. PCC 6803 did not show an alteration in growth at high salinity but continuously lost GG to the medium [61]. These experiments revealed that the compatible solute transporters among cyanobacteria are principally necessary to prevent leakage of these compounds from the cells to save organic carbon and energy. Recently, the genome of *Synechocystis* sp. PCC 6714 was published [63]. This strain is very closely related to *Synechocystis* sp. PCC 6803 but lacks the genes for the GG transporter. Correspondingly, cells of *Synechocystis* sp. PCC 6714 continuously lost GG into the medium and showed a lower salt tolerance limit than *Synechocystis* sp. PCC 6803 [63]. The existence of the GG transporter in *Synechocystis* sp. PCC 6803 also allowed experiments in which the defect in GG synthesis was complemented by the addition of external GG (Figure 2). As expected, GG supplementation allowed successful salt acclimation via the uptake of externally added GG [60] and proved that the accumulation of compatible solutes is sufficient for the reestablishment of cellular metabolism in salt-stressed cells [60,64]. Interestingly, the uptake of trehalose caused a decrease in the cellular content of previously synthesized GG [60]. This decrease indicates that a yet unknown pathway for degradation or modification of the endogenous GG pool may be involved in the salt acclimation of *Synechocystis* sp. PCC 6803.

Genes for GG-like transporters can be found in the genomes of many low and moderately halotolerant cyanobacteria, but have been not characterized yet. The genes for the glycine betaine transporter in halophilic strains are still unknown, however, the genomes of such strains code for transporters of the BCCT and ProU families that have been proved to be involved in glycine betaine transport among heterotrophic bacteria [54,65]. The further characterization of compatible solute transport among cyanobacteria will offer some interesting applications. First, using such transport activities one can provide externally cheap compatible solutes to support the growth of cyanobacteria in saline media. Second, if compatible solutes are to be harvested from cyanobacteria as high value products (discussed below), the corresponding transport activities can be knocked out to improve the extrusion of the product to the surrounding medium. Another possibility would offer the use of specific exporters for compatible solutes, such as CscB from *E. coli* for sucrose, which allowed a continuous sucrose production in salt-stressed cells of *Synechococcus elongatus* [66]. The excretion of GG has been described in stationary phase cells of the heterotrophic bacterium *Stenotrophomonas rhizophila* [67]. Based on its genome sequence, the salt-regulated gene expression of this bacterium was recently analyzed and revealed that the *ycaD* gene for a MFS-type transporter was co-expressed with the gene for GG synthesis [68]. Hence, this *ycaD* represents a likely gene candidate for the still unknown GG export channel, which could be expressed in GG-accumulating cyanobacteria to increase the yield of this compound.

## 3. Ion Transport

The cyanobacterial salt acclimation process is highly dynamic and includes the ion-mediated activation and/or inactivation of enzymes and transporters, as well as the global reprogramming of general gene expression profiles [69,70]. The export of Na^+^ is one of the fastest processes in salt-shocked cells. Immediately after addition of high amounts of NaCl, the cells rapidly shrink due to the immediate loss of water [71,72,73,74]. This shrinkage is reversed by the quick influx of external salts, such as Na^+^ and Cl^−^ [69], which allows water to return inside the cell. However, the high salt concentration in the cytoplasm inhibits cellular metabolism, including photosynthesis, transcription and particularly translation [70,75,76]. Therefore, the toxic Na^+^ is exchanged for the less toxic K^+^ [69]. Fast Na^+^ export is based on the activation of pre-existing ion-transport systems, such as different Na^+^/H^+^ antiporters [77,78,79] but likely also the more complex Mrp-system [80,81]. The halophilic strain *Aphanothece* *halophytica* exhibits a direct Na^+^-pumping ATPase that is most probably involved in the superior salt tolerance of this strain [82]. Subsequently, K^+^ and Cl^−^ are also mostly exported from the cells and replaced by the compatible solute GG [69]. The export of Cl^−^ out of the cyanobacterial cell is not well understood. There are several potential Cl^−^ channels and transporters annotated in cyanobacterial genomes. One of these channels from *Synechocystis* sp. PCC 6803 has been crystallized and it was proven to transport chloride albeit with rather low activity [83]. The transport of K^+^ is rather well investigated. In contrast to Na^+^ and Cl^−^ which are toxic toward cellular metabolism in high concentrations because they negatively influence protein structure, K^+^ is the main inorganic cation that contributes significantly to turgor generation. K^+^ is accumulated in cyanobacterial cells to approximately 150 mM and the amount accumulated is increased in salt-stressed cells. The salt-induced K^+^ transport is mainly driven by the Ktr system in *Synechocystis* sp. PCC 6803 because corresponding mutants showed a salt-sensitive phenotype [73,84] and were not able to restore cell volume shortly after salt shocks [74]. All cyanobacteria possess a high affinity K^+^ uptake system called Kdp, consisting of several subunits. The Kdp system is involved in the K^+^ uptake of salt or osmotically stressed enterobacteria [12]. This system has been intensively analyzed in *Anabaena* strains [85] and recently in *Synechocystis* sp. PCC 6803 [74]. These studies revealed that the cyanobacterial Kdp system has a very high affinity for K^+^. It most probably functions in K^+^ uptake during K^+^ limiting conditions, but it does not seem to play a significant role in acclimation to high salinity. However, the *kdp* genes are induced upon desiccation stress in *Nostoc* (*Anabaena*) sp. PCC 7120 and may support drought stress acclimation in cyanobacteria [86]. The concerted action of ion transporters is responsible for the maintenance of relatively low ion concentrations, which nearly correspond to the levels of cells grown at lower salinity.

## 4. Salt Stress and “omics”

**Figure 3:**
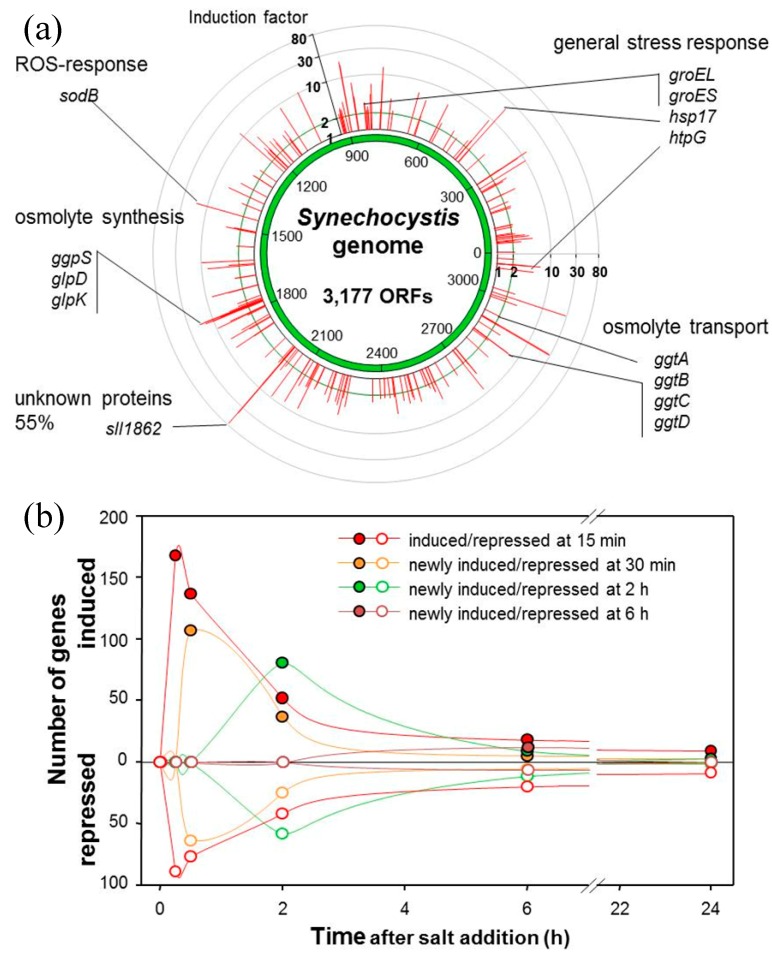
Global changes in the gene expression of *Synechocystis* sp. PCC 6803 after the addition of 4% NaCl to cells grown in freshwater medium. (**a**) Genes induced to different levels (red spikes represent about 200 genes) are shown on the chromosome of *Synechocystis*. Genes coding for proteins of special functions are highlighted. (**b**) The dynamic changes in the expression of different groups of genes are displayed. Genes are grouped in classes that show maximum expression or repression at selected time points after the addition of 4% NaCl (data from reference 70).

The term “omics” comprises technologies that focus on the collective characterization and quantification of pools of biological molecules to provide a valuable database for the molecular understanding of biological processes. Recently, transcriptomics and proteomics were applied to characterize global salt acclimation in several cyanobacterial strains including mutants. Transcriptomics was initially performed with DNA microarrays to investigate global gene expression changes in salt-stressed cells of the moderately halotolerant strain *Synechocystis* sp. PCC 6803 [70,87,88]. These studies revealed that 200–300 genes are up-regulated and about the same number is down-regulated after the addition of 4% NaCl (Figure 3). However, a time-series analysis showed that the majority of gene expression changes are rather transient. Particularly, genes showing an early response soon returned to the control expression level. Among the early responding genes, many code for proteins of unknown biological function. Only few genes stably remained at an enhanced expression level after long-term salt acclimation [70]. Many of the stable up-regulated genes code for proteins with a “meaningful” function regarding high salt acclimation. For example, genes that code for enzymes involved in the GG synthesis and transport belong to this group, but so do genes that code for general stress proteins known to stabilize protein structures as well those defending the cell against reactive oxygen species (ROS) such as *sodB* (Figure 3). In general, these datasets provided a comprehensive overview of the dynamic expression of all genes that are directly and indirectly involved in the cellular response of *Synechocystis* to salt stress conditions. Recently, the high-throughput sequencing of cDNA fragments (RNAseq) has become popular as a tool for transcriptomics. This technique not only allows quantification of transcripts but also provides information about transcriptional start sites, operon structures, and previously unannotated genes that code for small proteins or non-protein-coding (nc) RNAs, such as antisense RNAs or small regulatory RNAs. For *Synechocystis* sp. PCC 6803, RNAseq showed that nearly the same number of genes code for mRNAs as for ncRNAs, and the latter group of genes comprises some of the most highly expressed genes [89]. Moreover, these studies also revealed that the genome of cyanobacteria is highly dynamic because different growth conditions not only caused differential gene expression in terms of quantitative changes, it also revealed many new promoters and genes that became active only under specific environmental signals [63]. Regarding salt stress, the RNAseq approach was first applied to *Synechococcus* sp. PCC 7002 [90]. *Synechococcus*-cells treated at a rather high salinity of 1.5 M NaCl showed many gene expression changes, among them the accumulation of transcripts for genes coding for proteins involved in GG synthesis and transport, proteins for general stress response such as SodB, small high-light-induced proteins, flavoproteins, *etc.*, as was previously found in salt-shocked *Synechocystis* sp. PCC 6803. However, some differences were reported as well. For example, genes coding for ion transport proteins were stably induced in salt-shocked cells of *Synechococcus* sp. PCC 7002 and those for subunits of the photosystem 1, which were increased in *Synechocystis* sp. PCC 6803 under salt conditions [91], were found at rather decreased expression levels [90].

RNAseq was recently used to analyze expression changes in long-term (1–3 days) salt-acclimated cells of *Synechocystis* sp. PCC 6803 [92]. This study showed that many genes for protein biosynthesis and energy metabolism were found with decreased mRNA levels, and only a few genes showed increased levels, among them genes for fatty acid metabolism and a protein involved in CO_2_ acquisition. Mutants generated in selected genes with increased expression in salt-acclimated cells showed decreased growth in 4% NaCl, leading to the identification of new candidate proteins somehow involved in high salt acclimation [92]. Another study used RNAseq to compare the stress response of *Synechocystis* sp. PCC 6803 with *Synechococcus* *elongatus* [93]. Along with other environmental factors, the authors also analyzed the salt stress response. As expected for a comparison of a freshwater strain with a moderately halotolerant strain, there was only a small overlap in the transcriptional response to salt stress.

Changes in mRNA levels are not always translated into different protein abundances. Proteomics allows a quantitative overview of global changes in protein composition, however this technique is less representative than transcriptomics (usually only 20 maximally 50% of all proteins are detected). Initially, proteomics using 2-dimensional-gel-electrophoresis was used to compare the periplasmic [94], membrane [95] and soluble fractions [96] in salt-stressed and control cells of *Synechocystis* sp. PCC 6803. The same technology has been used to screen for salt-regulated proteins in the biotechnologically important cyanobacterium *Arthrospira platensis* [97]. These studies provided lists of many proteins that were enhanced after long-term salt acclimation. Among them were proteins for GG synthesis, and general stress proteins such as protein and RNA chaperones, SodB and thioredoxins. However, the proteomic approach also indicated that many hypothetical proteins were salt-regulated. Moreover, a corresponding increase in mRNA levels has not been reported for all of the proteins that are accumulated in salt-loaded cells [96]. This finding indicated that not only transcriptional control but also other regulatory levels such as protein stability contribute to the changed proteome of salt-acclimated cells. Recently, an integrated study on *Synechocystis* sp. PCC 6803 under salt stress conditions, coupling a quantitative proteomic and a RNAseq-based transcriptomic approach, was published [92]. Comparative quantification of protein abundance led to the identification of 68 and 108 proteins that are differentially regulated by salt-treatment at 24 and 48 h, respectively. The transcriptomic analysis showed that not all the genes were regulated in the same direction, *i.e.*, the authors also found cases in which the protein level increased and the mRNA level decreased. To obtain a deeper insight in the possible function of stress-regulated hypothetical proteins in *Synechocystis* sp. PCC 6803, gene expression was analyzed under 5 different conditions including high salt. The data set was grouped and revealed hypothetical proteins that seemed to react to all stresses, whereas others showed a more stress-specific pattern [98]. In addition to *Synechocystis* sp. PCC 6803, salt-induced alterations in the proteome were investigated in much detail with *Anabaena* strains of different salt tolerance. This study provided a comprehensive analysis of physiological and proteomic data in three different strains. The authors showed that even among closely related strains, distinct patterns of salt acclimation are present [99]. In *Nostoc* (*Anabaena*) sp. PCC 7120, the responses to salt and UV stress were compared. In addition to specific reactions, both stresses resulted in the accumulation of a defined set of common proteins. These proteins code for general stress proteins involved in the defense against ROS and the maintenance of DNA integrity as well as for many enzymes of sugar catabolism to provide more energy. However in general, salt stress induced many more proteins indicating that this stress is more harmful to this freshwater cyanobacterium than UV stress [100].

To our knowledge, no metabolome study of salt-treated cyanobacteria has been published as yet. It would be interesting to apply this “omics” technology because the accumulation of compatible solutes should have a dramatic impact on the overall primary metabolism. Especially for biotechnology, it will be important to know how and to what degree the carbon and nitrogen portioning inside the cyanobacterial cell changes in the presence of high salinity.

## 5. Sensing Salt Stress and Regulation of the Salt Acclimation Response

Transcriptomic and proteomic data show that multiple genes and proteins are differentially regulated in salt-stressed cells. These findings raise the question of how salt stress is sensed and how the signal is transduced to the target promoters. The search for the primary signal(s) after salt stress still has not provided a satisfying result. The most popular candidates are changes in the internal ion content and related changes in available water, which can result in altered membrane structure and tension or electrostatic effects influencing lipid-protein and protein-protein interactions [12,101]. In salt-shocked cyanobacterial cells, as in other microorganisms, the addition of a large amount of NaCl induces a rapid influx of different ions and the release of water (discussed in the section on ion export). These fluxes and changes in the cytoplasmic composition could possibly serve as a direct signal for the sensing of an acute salt stress situation. It has been shown that several proteins that fulfil crucial functions for basal salt acclimation are directly activated by an increasing NaCl content *in vitro*, whereas the majority of metabolic enzymes are inhibited in the presence of an elevated salt concentration. For example, the K^+^ transport system Ktr [73] and the key enzyme for GG synthesis GgpS [41,43] are directly activated by inorganic ions. For GgpS, the mechanism of NaCl-dependent activation has been analyzed in great detail [102]. At low salinity, the enzyme is present inside the cell but inactive due to its binding to the phosphate backbone of DNA and possibly RNA. This binding is sequence-independent and can be released by the addition of inorganic ions, preferentially KCl but also NaCl. Thus, the rapid influx of inorganic ions into salt-shocked cells of *Synechocystis* sp. PCC 6803 directly ensures the activation of the GG synthesis machinery. During the further salt acclimation, the internal ion content decreases, leading to a stepwise slowdown of GG accumulation. In fully salt-adapted cells, the increased GgpS level [103] resulting from the ion-mediated release of the small repressor protein GgpR from the *ggpS* promoter [104] and the salt-stress-proportional slight increase in Na^+^ and K^+^ guarantee a partially active GgpS pool that is sufficient to produce enough GG in coordination with growth under chronic salt stress [102]. Currently it is not clear if this elegant salt titration model also applies to other enzymes/proteins involved in salt acclimation.

However, there are indications that the ion content could also directly influence the gene expression machinery. For *E. coli*, it has been shown that the promoter selectivity of RNA polymerase changed depending on the K^+^-glutamate content. In the presence of increased amounts of K^+^-glutamate, which is accumulated in *E. coli* cells as a compatible solute in response to salt shock, the isolated RNA polymerase preferred promoters of genes that code for proteins crucial for the salt acclimation, such as those for the transporters of the compatible solutes [105]. The specificity of RNA polymerase could also be influenced via alternative sigma factors during the salt stress response in cyanobacteria. It has been shown that the expression of the *ggpS* gene is changed in the *sigF* mutant of *Synechocystis* sp. PCC 6803 leading to a decreased salt tolerance [103]. Similar results have been recently reported for the *Synechocystis* *sigB* mutant [106]. In addition to sigma factors SigF and SigB that are involved in the regulation of salt-specific genes such as *ggps*, all other group 2 sigma factors also might participate in high salt acclimation of the model cyanobacterium *Synechocystis* sp. PCC 6803. This notion is supported by the findings that single, double [107], and triple [108] mutants of group 2 sigma factors showed decreased growth in high salt stress. Some sigma factor genes (especially *sigB*) also have been detected among the genes differentially expressed in salt-stressed cells of *Synechocystis* sp. PCC 6803 [70,87,88]. Obviously, sigma factor cascades could be involved in the global reprogramming of the cyanobacterial metabolism to make it more tolerant against salt stress, which is a syndrome of many different single stress factors (discussed below in more detail).

There are additional promising candidates for the sensing and transducing salt stress. Two-component systems, composed of a sensory histidine kinase (Hik) and a corresponding response regulator (Rre) protein acting as a transcriptional activator/repressor, often play important roles in the sensing of chemical and physical signals from the environment in bacteria. Because such systems are involved in salt response of *E. coli*, a Hik mutant library of *Synechocystis* sp. PCC 6803 was screened to see if it shows changes in the global salt-induced transcriptome [88]. This study identified four histidine kinases, Hik16, Hik34, Hik33 and Hik41, that are somehow involved in the transduction of salt stress signals because approximately 20% of salt-inducible genes were not further salt-stimulated in these mutants. Subsequently, the corresponding Rre’s were identified [109]. However, none of the deregulated genes codes for proteins with salt-specific and/or essential functions such as *ggpS*. The majority of the affected genes code for proteins belonging to a large group of general stress proteins, such as proteins induced after ROS generation, high-light, or heat stress. It is also noteworthy that none of the investigated *hik* mutants showed decreased salt tolerance. Hik33, a putative salt-stress sensor, probably senses redox changes and was proposed to be located in the cyanobacterial thylakoid membrane. A *hik33* mutant was recently investigated regarding salt-induced changes in the proteome [110]. This study showed that a substantial rearrangement in the plasma membrane proteome occurs in *Synechocystis* sp. PCC 6803 after salt shock. However, other two-component systems still are promising candidates to study the regulation of genes essential for salt acclimation. A mutant of *Nostoc* (*Anabaena*) sp. PCC 7120 defective in the *rre* gene was found to be osmo-stress-sensitive [111]. This Rre, called OrrA, regulates the salt-stimulated sucrose synthesis. Mutants defective in *orrA* accumulated less sucrose, whereas the *orrA* overexpression resulted in higher sucrose levels [112]. Recently, a *slr1558* mutant of *Synechocystis* sp. PCC 6803 encoding for an OmpR-type Rre was found to be salt-sensitive and showed an altered expression of *ggpS* [113]. There are also reports indicating that protein phosphorylation and inositol monophosphate (IMP) could be involved in the signal transduction of the cyanobacterial salt stress response as has been reported for other organisms. Mutants with disrupted genes for the protein kinase SpkG in *Synechocystis* sp. PCC 6803 [114] and an IMP phosphatase of *Nostoc* (*Anabaena*) sp. PCC 7120 [115] showed decreased tolerances to salt and osmotic stress, respectively.

However, despite the many facets of potential mechanisms and players in the regulation of the salt stress response, a clear, unifying picture is not yet available. A possible reason for this difficulty might be that the acclimation toward salt stress comprises several specific responses to stresses such as high amounts of cations or anions, lack of water, membrane tension changes, increased ROS production due to a redox imbalance, *etc.* Thus, it will become very important to distinguish between salt-specific or more general stress responses and the involved signaling pathways. A better understanding of these mechanisms will also support the biotechnological use of cyanobacteria in saline systems. For example, it would be beneficial to couple product-specific enzymes to the salt response of the cells to increase the genetic stability of producer strains.

## 6. Salt Stress and Synthesis of Biotechnological Products

Developments regarding sustainable energy and new products beneficial to society are important factors to decrease the progress of climate change and to reduce the dependence on fossil fuels. Cyanobacteria harvest solar energy via photosynthesis and convert CO_2_ to organic matter. This ability has been previously used to design production strains for multiple biofuels or chemical feedstocks. The natural diversity of cyanobacteria includes species that show extreme environmental tolerance and are able to grow in areas inhospitable to crops. As briefly mentioned previously, future mass cultivation of cyanobacteria for biotechnology will certainly be performed in seawater [116], because freshwater resources are limited and must be used for drinking water and agricultural irrigation [117]. Therefore, optimal future producer strains must at least tolerate seawater conditions but likely even higher concentrations of salt because evaporation will quickly increase the external salt concentration in open cultivation systems. However, future cyanobacterial or microalgal cultivation for biotechnological applications is influenced by many additional environmental factors, such as temperature, light, nutrients, and pH [118,119,120]. Among the present widely used and well-analyzed model strains, *Synechococcus* sp. PCC 7002 seems to be the best suited for this purpose because it can grow well even in salt concentrations twice that of seawater [90], and the strain shows high resistance towards high light conditions and high temperatures [121].

Cultivation in saline media could also have beneficial effects in biofuel production. The autofermentation rates and the product yields could be significantly enhanced by “sodium stress cycling” [122]. This strategy combines two cultivation steps. First, the cultivation of *Arthrospira maxima* under high salt conditions was used to achieve an increased carbohydrate content that served as internal compatible solutes. Second, the salt-grown cells were exposed to a hypoosmotic stress to drive catabolism of the previously accumulated carbohydrate reserves during autofermentation. This combination strategy resulted in a 121-fold higher yield of ethanol production at a high salt concentration compared to a lower salt concentration [122]. However, the compatible solutes sucrose and GG were found to be less effective substrates for autofermentation than glycogen in the cyanobacterium *Synechococcus* sp. PCC 7002 [123]. The compatible solute itself may be an interesting (by)product from cyanobacterial cells cultivated at high salinity, because these compounds have interesting biological features [34,35,40]. These compounds are sold as extremolytes for pharmaceutical and cosmetic applications [124]. Thus, cyanobacteria could be used as “cellular factories” for the production of compatible solutes that are accumulated in response to salt stress. Recently, an efficient production system for sucrose was described using the cyanobacterium *Synechococcus elongates* PCC 7942 [66]. Sucrose is a compatible solute that is naturally synthesized (as mentioned above) by many cyanobacteria. The great advantage of a biotechnological production system for sugars such as sucrose is the accumulation of this compound in high concentrations (molar) in the cytoplasm of the cyanobacteria. The expression of an effective export system such as a sucrose permease allows the continuous excretion of sucrose into the medium [66]. Then, the biotechnologically interesting compound can be easily and cost-efficiently removed from the growth medium because cell harvesting and subsequent extraction can represent up to 30% of the total production costs [125]. Recently, sucrose production was tested with additional cyanobacterial strains. Beside the expression of the sucrose transporter CscB, mutations in competing reactions such as GG synthesis and the overexpression of the sucrose synthesis genes resulted in a considerable increase in the sucrose yield [126]. As an alternative to the expression of sugar export proteins, compatible solutes can be easily harvested from salt-acclimated cells after transfer into hypoosmotic media. Under such conditions, the compatible solute pool is almost completely released into the medium [127,128] to avoid lysing the cells. After immobilization of the cells in alginate, salt-acclimation and hypoosmotic harvest cycles can be repeated several times, resulting in quite high yields of valuable products [127,129]. Another interesting product from cyanobacteria and microalgae are lipids that can be used as biodiesel. It is known that the lipid content and the lipid composition in cells are influenced by salt stress. Increased salt concentrations raised the lipid content from 60% to 70% in the green algae *Dunaliella tertiolecta* ATCC 30929 [130]. Another strategy to improve the yield of biofuels might be a combination of multiple stress factors. The total lipid content of the microalga *Nannochloropsis* sp. was increased by simultaneously applying high light stress and high salinity stress [131]. Thus, in addition to the negative influence of high salt concentrations on growth efficiency, cultivation under these conditions might also have positive effects on the amount of product, spectrum and fermentation duration.

Another application strategy is using the knowledge about cyanobacterial salt stress acclimation to generate more tolerant producer strains for biofuel production. Many genes that are involved in the stress response are well known and can be transferred into less tolerant organisms. The glycine methylation pathway for glycine betaine biosynthesis from *A. halophytica* was transferred and expressed in *Synechococcus elongates,* leading to glycine betaine accumulation and increased salt tolerance. It also could be expressed in the model plant Arabidopsis, where it also improved the growth on salt media [132]. Genes for GG biosynthesis were also transferred into Arabidopsis [133] and later into potato [134], which slightly improved the salt tolerance of the transgenic plants. In addition to genes for compatible solutes, those for general stress proteins might also be useful. Recently, a *Synechocystis* strain overexpressing the *sigB* gene has been generated. This strain not only showed increased temperature tolerance, it was also resistant to rather high amounts of the potential biofuel butanol [135]. Another example concerns genes for proper redox regulation, for example, genes coding for a glutathione peroxidase or an ascorbate peroxidase that are involved in scavenging reactive oxygen species (ROS). It has been reported that overexpression of these enzymes improved tolerance to high light stress, low temperature stress and high salinity stress in transgenic plants [136,137]. Such attempts could also increase the stress tolerance of the cyanobacterial producer and allow cultivation in high salt media, which also prevents the proliferation of invasive species in the photobioreactor.

## 7. Conclusions

The evolution of effective salt-acclimation mechanisms allowed cyanobacteria to colonize habitats that had low to high salt concentrations. For model cyanobacteria, the acclimation strategy is well characterized on the physiological and molecular levels. Three salt-tolerant groups, freshwater, moderately salt-tolerant and halophilic cyanobacteria, have been defined, which differ not only in their ultimate salinity tolerance levels but also in the chemical nature of the major compatible solute. However, among moderately halotolerant cyanobacteria that accumulate GG, marked differences in the maximum salt tolerance were found. This finding indicates that additional processes such as an efficient ion export system contribute significantly to salt resistance. The energetic costs of salt acclimation must be better analyzed because continuous ion pumping, in particular, will limit higher yields of valuable products from salt-stressed cyanobacterial cells. Nevertheless, the accumulated knowledge of salt acclimation from model cyanobacteria can be transferred to cyanobacteria that are not well studied. For example, many cyanobacterial genomes are available in public data bases and could be searched for key genes coding proteins for essential salt acclimation processes such as compatible solute biosynthesis and ion export. However, the application of modern “omics” technologies has revealed that many more genes are clearly salt-regulated. Among them, genes for multiple general stress proteins such as chaperones or enzymes defending the cell against ROS, and participating in cell wall and membrane reorganization have been found. Unfortunately, the signaling and regulation of the cyanobacterial salt stress response is not currently well understood. A possible reason is that salt stress is a highly complex situation characterized by ionic and osmotic stresses and many secondary stresses, such as oxidative stress. Therefore, it is difficult to distinguish between the response to general stress or to the specific salt stress situation. Many players regulating single genes/proteins have been identified and will be used to obtain a more integrated view of salt stress regulation in the near future. A comprehensive understanding of the entire salt-acclimation process would be of great value for the future biotechnological application of cyanobacteria as chemical feedstock and for the production of biofuels or high-value products. Mass cultivation of cyanobacteria for such purposes must be performed using saline water to avoid competition with scarce freshwater resources. Thus, species naturally competent to grow at high salinity that are also light and temperature tolerant will become the most promising starting points to develop cyanobacterial producer strains. We are now equipped with much knowledge, as well as a genetic and analytical toolbox to manipulate and analyze metabolic pathways with high precision. The application of these tools will further improve the application of cyanobacteria and will allow their development as highly efficient and cost-effective production systems as sources of renewable fuel and chemical feedstocks.

## References

[1] Tandeau de Marsac N.T., Houmard J. (1993). Adaptation of cyanobacteria to environmental stimuli: New steps toward molecular mechanisms. FEMS Microbiol. Rev..

[2] Oren A., Whitton B.A., Potts M. (2000). Salts and brines. The Ecology of Cyanobacteria.

[3] Ladas N.P., Papageorigou G.C. (2000). Cell turgor: A critical factor for the proliferation of cyanobacteria at unfavorable salinity. Photosynth Res..

[4] Galinski E.A., Trüper H.G. (1994). Microbial behaviour in salt-stressed ecosystems. FEMS Microbiol. Rev..

[5] Hagemann M. (2011). Molecular biology of cyanobacterial salt acclimation. FEMS Microbiol. Rev..

[6] Oren A., Balows A., Trüper H.G., Dworkin M., Harder W., Schleifer K.-H. (1993). The genera Haloanaerobium, Halobacteroides and Sporohalobacter. The Prokaryotes.

[7] Oren A., Mana L. (2002). Amino acid composition of bulk protein and salt relationships of selected enzymes of *Salinibacter ruber*, an extremely halophilic bacterium. Extremophiles.

[8] Oren A. (1999). Bioenergetic aspects of halophilism. Microbiol. Mol. Biol. Rev..

[9] Müller V., Oren A. (2003). Metabolism of chloride in halophilic prokaryotes. Extremophiles.

[10] Brown A.D. (1976). Microbial water stress. Bacteriol. Rev..

[11] Bremer E., Krämer R., Hengge-Aronis R. (2000). Coping with osmotic challenges: osmoregulation through accumulation and release of compatible solute in bacteria. Bacterial Stress Responses.

[12] Wood J. (2011). Bacterial osmoregulation: a paradigm for the study of cellular homeostasis. Annu. Rev. Microbiol..

[13] Krämer R. (2010). Bacterial stimulus perception and signal transduction: Response to osmotic stress. Chem. Rec..

[14] Kempf B., Bremer E. (1998). Uptake and synthsis of compatible solutes as microbial stress response to high-osmolality environment. Arch. Microbiol..

[15] Potts M. (1994). Desiccation tolerance of prokaryotes. Microbiol. Rev..

[16] Klähn S., Hagemann M. (2011). Compatible solute biosynthesis in cyanobacteria. Environ. Microbiol..

[17] Hagemann M., Chauvat F., Cassier-Chauvat C. (2013). Genomics of salt acclimation: synthesis of compatible solutes among cyanobacteria. Genomics of Cyanobacteria.

[18] Pade N., Hagemann M., Srivastava A.K., Rai A.N., Neilan B.A. (2013). Cyanobacterial salt stress acclimation: genetic manipulation and regulation. Stress Biology of Cyanobacteria: Molecular Mechanisms to Cellular Responses.

[19] Arakawa T., Timasheff S.N. (1985). The stabilization of proteins by osmolytes. Biophys. J..

[20] Wood J.M. (1999). Osmosensing by bacteria: Signals and membrane-based sensors. Micobiol. Mol. Biol. Rev..

[21] Poolman B., Glaasker E. (1998). Regulation of compatible solute accumulation in bacteria. Mol. Microbiol..

[22] Reed R.H., Borowitzka L.J., Mackay M.A., Chudek J.A., Foster R., Warr S.R.C., Moore D.J., Stewart W.D.P. (1986). Organic solute accumulation in osmotically stressed cyanobacteria. FEMS Microbiol. Lett..

[23] Mackay M.A., Norton R.S., Borowitzka L.J. (1984). Organic osmoregulatory solutes in cyanobacteria. J. Gen. Microbiol..

[24] Singh A.K., Chakarvathy D., Singh T.P.K., Singh H.N. (1996). Evidence for a role for l-proline as a salinity protectant in the cyanobacterium *Nostoc muscorum*. Plant Cell Environ..

[25] Fulda S., Huckauf J., Schoor A., Hagemann M. (1999). Analysis of stress responses in the cyanobacterial strains *Synechococcus* sp. PCC 7942, *Synechocystis* sp. PCC 6803, and *Synechococcus* sp. PCC 7418: Osmolyte accumulation and stress proteins synthesis. J. Plant Physiol..

[26] Klähn S., Steglich C., Hess W.R., Hagemann M. (2010). Glucosylglycerate: A secondary compatible solute common to marine cyanobacteria from nitrogen-poor environments. Environ. Microbiol..

[27] Shih P.M., Wu D., Latifi A., Axen S.D., Fewer D.P., Talla E., Calteau A., Cai F., Tandeau de Marsac N., Rippka R. (2013). Improving the coverage of the cyanobacterial phylum using diversity-driven genome sequencing. Proc. Natl. Acad. Sci. USA.

[28] Hewson I., Poretsky R.S., Beinart R.A., White A.E., Shi T., Bench S.R., Moisander P.H., Paerl R.W., Tripp H.J., Montoya J.P., Moran M.A., Zehr J.P. (2009). *In situ* transcriptomic analysis of the globally important keystone N_2_-fixing taxon *Crocosphaera watsonii*. ISME J.

[29] Pade N., Compaore J., Klähn S., Stal L.J., Hagemann M. (2012). The marine cyanobacterium *Crocosphaera watsonii* WH8501 synthesizes the compatible solute trehalose by a laterally acquired OtsAB fusion protein. Environ. Microbiol..

[30] Bergman B., Sandh G., Lin S., Larsson J., Carpenter E.J. (2013). *Trichodesmium*—a widespread marine cyanobacterium with unusual nitrogen fixation properties. FEMS Microbiol. Rev..

[31] Salerno G.L., Porchia A.C., Vargas W.A., Abdian P.L. (2004). Fructose-containing oligosaccharides: Novel compatible solutes in *Anabaena* cells exposed to salt stress. Plant Sci..

[32] Fischer D., Geyer A., Loos E. (2006). Occurrence of glucosylsucrose [α-d-glucopyranosyl-(1→2)-α-d-glucopyranosyl-(1→2)-β-d-fructofuranoside] and glucosylated homologues in cyanobacteria: Structural properties, cellular contents and possible function as thermoprotectants. FEBS J..

[33] Goh F., Barrow K.D., Burns B.P., Neilan B.A. (2010). Identification and regulation of novel compatible solutes from hypersaline stromatolite-associated cyanobacteria. Arch. Microbiol..

[34] Borges N., Ramos A., Raven N.D.H., Sharp R.J., Santos H. (2002). Comparative study of the thermostabilizing properties of mannosylglycerate and other compatible solutes on model enzymes. Extremophiles.

[35] Hincha D.K., Hagemann M. (2004). Stabilization of model membranes during drying by compatible solutes involved in the stress tolerance of plants and microorganisms. Biochem. J..

[36] Kandror O., DeLeon A., Goldberg A.L. (2002). Trehalose synthesis is induced upon exposure of *Escherichia coli* to cold and is essential for viability at low temperatures. Proc. Natl. Acad. Sci. USA.

[37] Jang I.C., Oh S.J., Seo J.S., Choi W.B., Song S.I., Kim C.H., Kim Y.S., Seo H.S., Choi Y.D., Nahm B.H. (2003). Expression of a bifunctional fusion of the *Escherichia coli* genes for trehalose-6-phosphate synthase and trehalose-6-phosphate phosphatase in transgenic rice plants increases trehalose accumulation and abiotic stress tolerance without stunting growth. Plant Physiol..

[38] Diamant S., Rosenthal D., Azem A., Eliahu N., Ben-Zvi A.P., Goloubinoff P. (2003). Dicarboxylic amino acids and glycine-betaine regulate chaperone-mediated protein-disaggregation under stress. Mol. Microbiol..

[39] Sawangwan T., Goedl C., Nidetzky B. (2010). Glucosylglycerol and glucosylglycerate as enzyme stabilizers. Biotechnol. J..

[40] Luley-Goedel C., Nidetzky B. (2011). Glycosides as compatible solutes: biosynthesis and applications. Nat. Prod. Rep..

[41] Hagemann M., Erdmann N. (1994). Activation and pathway of glucosylglycerol biosynthesis in the cyanobacterium *Synechocystis* sp. PCC 6803. Microbiology.

[42] Hagemann M., Schoor A., Jeanjean R., Zuther E., Joset F. (1997). The *stpA* gene form *Synechocystis* sp. strain PCC 6803 encodes the glucosylglycerol-phosphate phosphatase involved in cyanobacterial osmotic response to salt shock. J. Bacteriol..

[43] Marin K., Zuther E., Kerstan T., Kunert A., Hagemann M. (1998). The *ggpS* gene from *Synechocystis* sp. strain PCC 6803 encoding glucosylglycerol-phosphate synthase is involved in osmolyte synthesis. J. Bacteriol..

[44] Porchia A.C., Salerno G.L. (1996). Sucrose biosynthesis in a prokaryotic organism: Presence of two sucrose-phosphate synthases in *Anabaena* with remarkable differences compared with the plant enzymes. Proc. Natl. Acad. Sci. USA.

[45] Higo A., Katoh H., Ohmori K., Ikeuchi M., Ohmori M. (2006). The role of a gene cluster for trehalose metabolism in dehydration tolerance of the filamentous cyanobacterium *Anabaena* sp. PCC 7120. Microbiology.

[46] Waditee R., Tanaka Y., Aoki K., Hibino T., Jikuya H., Takabe T., Takabe T. (2003). Isolation and functional characterization of N-methyltransferase that catalyze betaine synthesis from glycine in a halotolerant photosynthetic organism *Aphanothece halophytica*. J. Biol. Chem..

[47] Martínez-Noël G.M., Cumino A.C., Kolman M.A., Salerno G.L. (2013). First evidence of sucrose biosynthesis by single cyanobacterial bimodular proteins. FEBS Lett..

[48] Kolman M.A., Torres L.L., Martin M.L., Salerno G.L. (2011). Sucrose synthase in unicellular cyanobacteria and its relationship with salt and hypoxic stress. Planta.

[49] Azua-Bustos A., Zúñiga J., Arenas-Fajardo C., Orellana M., Salas L., Rafael V. (2014). *Gloeocapsopsis* AAB1, an extremely desiccation-tolerant cyanobacterium isolated from the Atacama Desert. Extremophiles.

[50] Yoshikawa T., Ikeda Y., Sakata T., Maeda H. (2011). Cloning and analysis of the *ggpS* gene from Cyanobacteria *Arthrospira* spp. involved in the synthesis of an osmolyte glucosylglycerol. Biocontrol. Sci..

[51] Kothari A., Vaughn M., Garcia-Pichel F. (2013). Comparative genomic analyses of the cyanobacterium, *Lyngbya aestuarii* BL J, a powerful hydrogen producer. Front. Microbiol..

[52] Mao X., Olman V., Stuart R., Paulsen I.T., Palenik B., Xu Y. (2010). Computational prediction of the osmoregulation network in *Synechococcus* sp. WH8102. BMC Genomics.

[53] Csonka L.N., Hanson A.D. (1991). Prokaryotic osmoregulation: genetics and physiology. Annu. Rev. Microbiol..

[54] Ziegler C., Bremer E., Krämer R. (2010). The BCCT family of carriers: from physiology to crystal structure. Mol. Microbiol..

[55] Pittelkow M., Bremer E., Ventosa A., Ohren A., Ma Y. (2011). Cellular adjustment of *Bacillus subtilis* and other bacilli to fluctuating salinities. Halophiles and Hypersaline Environments: Current Research and Future Trends.

[56] Lucht J.M., Bremer E. (1995). Adaptation of *Escherichia coli* to high osmolarity environments: Osmoregulation of the high-affinity glycine betaine transport system *ProU*. FEMS Microbiol. Rev..

[57] Moore D.J., Reed R.H., Stewart W.D.P. (1987). A glycine betaine transport system in *Aphanothece*
*halophytica* and other glycine betaine-synthesizing cyanobacteria. Arch. Microbiol..

[58] Laloknam S., Tanaka K., Buaboocha T., Waditee R., Incharoensakdi A., Hibino T., Tanaka Y., Takabe T. (2006). Halotolerant cyanobacterium *Aphanothece halophytica* contains a betaine transporter active at alkaline pH and high salinity. Appl. Environ. Microbiol..

[59] Mikkat S., Hagemann M., Schoor A. (1996). Active transport of glucosylglycerol is involved in salt adaptation of the cyanobacterium *Synechocystis* sp. PCC 6803. Arch. Microbiol..

[60] Mikkat S., Effmert U., Hagemann M. (1997). Uptake and use of the osmoprotective compounds trehalose, glucosylglycerol, and sucrose by the cyanobacterium *Synechocystis* sp. PCC6803. Arch. Microbiol..

[61] Hagemann M., Richter S., Mikkat S. (1997). The *ggtA* gene encodes a subunit of the transport system for the osmoprotective compound glucosylglycerol in *Synechocystis* sp. strain PCC 6803. J. Bacteriol..

[62] Mikkat S., Hagemann M. (2000). Molecular analysis of the *ggtBCD* gene cluster of *Synechocystis* sp. Strain PCC6803 encoding subunits of an ABC transporter for osmoprotective compounds. Arch. Microbiol.

[63] Kopf M., Klähn S., Pade N., Weingärtner C., Hagemann M., Voß B., Hess W.R. (2014). Comparative genome analysis of the closely related *Synechocystis* strains PCC 6714 and PCC 6803. DNA Res..

[64] Ferjani A., Mustardy L., Sulpice R., Marin K., Suzuki I., Hagemann M., Murata N. (2003). Glucosylglycerol, a compatible solute, sustains cell division under salt stress. Plant Physiol..

[65] Horn C., Jenewein S., Sohn-Bösser L., Bremer E., Schmitt L. (2005). Biochemical and structural analysis of the *Bacillus subtilis* ABC transporter OpuA and its isolated subunits. J. Mol. Microbiol. Biotechnol..

[66] Ducat D.C., Avelar-Rivas J.A., Way J.C., Silver P.A. (2012). Rerouting carbon flux to enhance photosynthetic productivity. Appl. Environ. Microbiol..

[67] Roder A., Hoffmann E., Hagemann M., Berg G. (2005). Synthesis of the compatible solutes glucosylglycerol and trehalose by salt-stressed cells of *Stenotrophomonas* strains. FEMS Microbiol. Lett..

[68] Alavi P., Starcher M.R., Zachow C., Müller H., Berg G. (2013). Root-microbe systems: The effect and mode of interaction of stress protecting agent (SPA) *Stenotrophomonas rhizophila* DSM14405^T^. Front. Plant Sci..

[69] Reed R.H., Warr S.R.C., Richardson D.L., Moore D.J., Stewart W.D.P. (1985). Multiphasic osmotic adjustment in a euryhaline cyanobacterium. FEMS Microbiol. Lett..

[70] Marin K., Kanesaki Y., Los D.A., Murata N., Suzuki I., Hagemann M. (2004). Gene expression profiling reflects physiological processes in salt acclimation of *Synechocystis* sp. Strain PCC 6803. Plant Physiol..

[71] Blumwald E., Mehlhorn R.J., Packer L. (1983). Studies of osmoregulation in salt adaptation of cyanobacteria with ESR spin-probe techniques. Proc. Natl. Acad. Sci. USA.

[72] Shapiguzov A., Lyukevich A.A., Allakhverdiev S.I., Sergeyenko T.V., Suzuki I., Murata N., Los D.A. (2005). Osmotic shrinkage of cells of *Synechocystis* sp. PCC 6803 by water efflux via aquaporins regulates osmostress-inducible gene expression. Microbiology.

[73] Matsuda N., Kobayashi H., Katoh H., Ogawa T., Futatsugi L., Nakamura T., Bakker E.P., Uozumi N. (2004). Na^+^-dependent K^+^ uptake Ktr system from the cyanobacterium *Synechocystis* sp. PCC 6803 and its role in the early phases of cell adaptation to hyperosmotic shock. J. Biol. Chem..

[74] Nanatani K., Shijuku T., Takano Y., Zulkifli L., Yamazaki T., Tominaga A., Souma S., Onai K., Morishita M., Ishiura M. (2014). Comparative analysis of *kdp* and *ktr* mutants reveals distinct doles of the potassium transporters in the model cyanobacterium *Synechocystis* sp. PCC 6803. J. Bacteriol..

[75] Schubert H., Fulda S., Hagemann M. (1993). Effects of adaptation to different salt concentrations on photosynthesis and pigmentation of the cyanobacterium *Synechocystis* sp. PCC 6803. J. Plant Physiol..

[76] Hagemann M., Fulda S., Schubert H. (1994). DNA, RNA and protein synthesis in the cyanobacterium *Synechocystis* sp. PCC 6803 adapted to different salt concentrations. Curr. Microbiol..

[77] Elanskaya I.V., Karandashova I.V., Bogachev A.V., Hagemann M. (2002). Functional analysis of the Na^+^/H^+^ antiporter encoding genes of the cyanobacterium *Synechocystis* PCC 6803. Biochemistry (Moscow).

[78] Wang H.L., Postier B.L., Burnap R.L. (2002). Polymerase chain reaction-based mutageneses identify key transporters belonging to multigene families involved in Na^+^ and pH homeostasis of *Synechocystis* sp. PCC 6803. Mol. Microbiol..

[79] Tsunekawa K., Shijuku T., Hayashimoto M., Kojima Y., Onai K., Morishita M., Ishiura M., Kuroda T., Nakamura T., Kobayashi H. (2009). Identification and characterization of the Na^+^/H^+^ antiporter Nhas3 from the thylakoid membrane of *Synechocystis* sp. PCC 6803. J. Biol. Chem..

[80] Blanco-Rivero A., Leganés F., Fernández-Valiente E., Calle P., Fernández-Piñas F. (2005). *mrpA*, a gene with roles in resistance to Na^+^ and adaptation to alkaline pH in the cyanobacterium *Anabaena* sp. PCC7120. Microbiology.

[81] Fukaya F., Promden W., Hibino T., Tanaka Y., Nakamura T., Takabe T. (2009). An Mrp-like cluster in the halotolerant cyanobacterium *Aphanothece halophytica* functions as a Na^+^/H^+^ antiporter. Appl. Environ. Microbiol..

[82] Soontharapirakkul K., Promden W., Yamada N., Kageyama H., Incharoensakdi A., Iwamoto-Kihara A., Takabe T. (2011). Halotolerant cyanobacterium *Aphanothece*
*halophytica* contains an Na^+^-dependent F1F0-ATP synthase with a potential role in salt-stress tolerance. J. Biol. Chem..

[83] Jayaram H., Robertson J.L., Wu F., Williams C., Miller C. (2011). Structure of a slow CLC Cl^−^/H^+^ antiporter from a cyanobacterium. Biochemistry.

[84] Berry S., Esper B., Karandashova I., Teuber M., Elanskaya I., Rögner M., Hagemann M. (2003). Potassium uptake in the unicellular cyanobacterium *Synechocystis* sp. strain PCC 6803 mainly depends on a Ktr-like system encoded by *slr*1509 (*ntpJ*). FEBS Lett..

[85] Ballal A., Basu B., Apte S.K. (2007). The Kdp-ATPase system and its regulation. J. Biosci..

[86] Katoh H., Asthana R.K., Ohmori M. (2004). Gene expression in the cyanobacterium *Anabaena* sp. PCC7120 under desiccation. Microb. Ecol..

[87] Kanesaki Y., Suzuki I., Allakhverdiev S.I., Mikami K., Murata N. (2002). Salt stress and hyperosmotic stress regulate the expression of different sets of genes in *Synechocystis* sp. PCC 6803. Biochem. Biophys. Res. Commun..

[88] Marin K., Suzuki I., Yamaguchi K., Ribbeck K., Yamamoto H., Kanesaki Y., Hagemann M., Murata N. (2003). Identification of histidine kinases that act as sensors in the perception of salt stress in *Synechocystis* sp. PCC 6803. Proc. Natl. Acad. Sci. USA.

[89] Mitschke J., Georg J., Scholz I., Sharma C.M., Dienst D., Bantscheff J., Voss B., Steglich C., Wilde A., Vogel J., Hess W.R. (2011). An experimentally anchored map of transcriptional start sites in the model cyanobacterium *Synechocystis* sp. PCC6803. Proc. Natl Acad Sci USA.

[90] Ludwig M., Bryant D.A. (2012). *Synechococcus* sp. strain PCC 7002 transcriptome: acclimation to temperature, salinity, oxidative stress, and mixotrophic growth conditions. Front. Microbiol..

[91] Schubert H., Hagemann M. (1990). Salt effect on 77 K fluorescence and photosynthesis in the cyanobacterium *Synechocystis* spec. PCC 6803. FEMS Microbiol. Lett..

[92] Qiao J., Huang S., Te R., Wang J., Chen L., Zhang W. (2013). Integrated proteomic and transcriptomic analysis reveals novel genes and regulatory mechanisms involved in salt stress responses in *Synechocystis* sp. PCC 6803. Appl. Microbiol. Biotechnol..

[93] Billis K., Billini M., Tripp H.J., Kyrpides N.C., Mavromatis K. (2014). Comparative transcriptomics between *Synechococcus* PCC 7942 and *Synechocystis* PCC 6803 provide insights into mechanisms of stress acclimation. PLoS One.

[94] Fulda S., Huang F., Nilsson F., Hagemann M., Norling B. (2000). Proteomics of *Synechocystis* sp. strain PCC 6803 identification of periplasmic proteins in cells grown at low and high salt concentrations. Eur. J. Biochem..

[95] Huang F., Fulda S., Hagemann M., Norling B. (2006). Proteomic screening of salt-stress-induced changes in plasma membranes of *Synechocystis* sp. strain PCC 6803. Proteomics.

[96] Fulda S., Mikkat S., Huang F., Huckauf J., Marin K., Norling B., Hagemann M. (2006). Proteome analysis of salt stress response in the cyanobacterium *Synechocystis* sp. strain PCC 6803. Proteomics.

[97] Wang H., Yang Y., Chen W., Ding L., Li P., Zhao X., Wang X., Li A., Bao Q. (2013). Identification of differentially expressed proteins of *Arthrospira* (*Spirulina*) *platensis*-YZ under salt-stress conditions by proteomics and qRT-PCR analysis. Proteome Sci..

[98] Qiao J., Shao M., Chen L., Wang J., Wu G., Tian X., Liu J., Huang S., Zhang W. (2013). Systematic characterization of hypothetical proteins in *Synechocystis* sp. PCC 6803 reveals proteins functionally relevant to stress responses. Gene.

[99] Rai S., Agrawal C., Shrivastava A.K., Singh P.K., Rai L.C. (2014). Comparative proteomics unveils cross species variations in *Anabaena* under salt stress. J. Proteomics.

[100] Rai S., Singh S., Shrivastava A.K., Rai L.C. (2013). Salt and UV-B induced changes in *Anabaena* PCC 7120: Physiological, proteomic and bioinformatic perspectives. Photosynth. Res..

[101] Poolman B., Spitzer J.J., Wood J.M. (2004). Bacterial osmosensing: roles of membrane structure and electrostatics in lipid-protein and protein-protein interactions. Biochim. Biophys. Acta.

[102] Novak J.F., Stirnberg M., Roenneke B., Marin K. (2011). A novel mechanism of osmosensing, a salt-dependent protein-nucleic acid interaction in the cyanobacterium *Synechocystis* species PCC 6803. J. Biol Chem.

[103] Marin K., Huckauf J., Fulda S., Hagemann M. (2002). Salt-dependent expression of glucosylglycerol-phosphate synthase, involved in osmolyte synthesis in the cyanobacterium *Synechocystis* sp. strain PCC 6803. J. Bacteriol..

[104] Klähn S., Höhne A., Simon E., Hagemann M. (2010). The gene *ssl3076* encodes a protein mediating the salt-induced expression of *ggpS* for the biosynthesis of the compatible solute glucosylglycerol in *Synechocystis* sp. strain PCC 6803. J. Bacteriol..

[105] Gralla J.D., Vargas D.R. (2006). Potassium glutamate as a transcriptional inhibitor during bacterial osmoregulation. EMBO J..

[106] Nikkinen H.L., Hakkila K., Gunnelius L., Huokko T., Pollari M., Tyystjärvi T. (2012). The SigB σ factor regulates multiple salt acclimation responses of the cyanobacterium *Synechocystis* sp. PCC 6803. Plant Physiol..

[107] Pollari M., Gunnelius L., Tuominen I., Ruotsalainen V., Tyystjärvi E., Salminen T., Tyystjärvi T. (2008). Characterization of single and double inactivation strains reveals new physiological roles for group 2 sigma factors in the cyanobacterium *Synechocystis* sp. PCC 6803. Plant Physiol..

[108] Tyystjärvi T., Huokko T., Rantamäki S., Tyystjärvi E. (2013). Impact of different group 2 sigma factors on light use efficiency and high salt stress in the cyanobacterium *Synechocystis* sp. PCC 6803. PLoS One.

[109] Shoumskaya M.A., Paithoonrangsarid K., Kanesaki Y., Los D.A., Zinchenko V.V., Tanticharoen M., Suzuki I., Murata N. (2005). Identical Hik-Rre systems are involved in perception and transduction of salt signals and hyperosmotic signals but regulate the expression of individual genes to different extents in *Synechocystis*. J. Biol. Chem..

[110] Li T., Yang H.M., Cui S.X., Suzuki I., Zhang L.F., Li L., Bo T.T., Wang J., Murata N., Huang F. (2012). Proteomic study of the impact of Hik33 mutation in *Synechocystis* sp. PCC 6803 under normal and salt stress conditions. J. Proteome Res..

[111] Schwartz S.H., Black T.A., Jäger K., Panoff J.M., Wolk C.P. (1998). Regulation of an osmoticum-responsive gene in *Anabaena* sp. strain PCC 7120. J. Bacteriol..

[112] Ehira S., Kimura S., Miyazaki S., Ohmori M. (2014). Sucrose synthesis in the nitrogen-fixing cyanobacterium *Anabaena* sp. strain PCC 7120 is controlled by the two-component response regulator OrrA. Appl. Environ. Microbiol..

[113] Chen L., Wu L., Zhu Y., Song Z., Wang J., Zhang W. (2014). An orphan two-component response regulator Slr1588 involves salt tolerance by directly regulating synthesis of compatible solutes in photosynthetic *Synechocystis* sp. PCC 6803. Mol. Biosyst..

[114] Liang C., Zhang X., Chi X., Guan X., Li Y., Qin S., Shao H.B. (2011). Serine/threonine protein kinase SpkG is a candidate for high salt resistance in the unicellular cyanobacterium *Synechocystis* sp. PCC 6803. PLoS One.

[115] Wang F.K., Latifi A., Chen W.L., Zhang C.C. (2012). The inositol monophosphatase All2917 (IMPA1) is involved in osmotic adaptation in *Anabaena* sp. PCC7120. Environ. Microbiol. Rep..

[116] Algenol Biofuels. http://www.algenol.com/.

[117] Chisti Y. (2013). Constraints to commercialization of algal fuels. J. Biotechnol..

[118] Guschina I.A., Harwood J.L. (2006). Lipids and lipid metabolism in eukaryotic algae. Prog. Lipid Res..

[119] Hu Q., Sommerfeld M., Jarvis E., Ghirardi M., Posewitz M., Seibert M., Darzins A. (2008). Microalgal triacylglycerols as feedstocks for biofuel production: perspectives and advances. Plant J..

[120] Singh N.K., Dhar D.W. (2011). Microalgae as second generation biofuel. A review. Agron. Sustain. Dev..

[121] Bernstein H.C., Konopka A., Melnicki M.R., Hill E.A., Kucek L.A., Zhang S., Shen G., Bryant D.A., Beliaev A.S. (2014). Effect of mono- and dichromatic light quality on growth rates and photosynthetic performance of *Synechococcus* sp. PCC 7002. Front. Microbiol..

[122] Carrieri D., Momot D., Brasg I.A., Ananyev G., Lenz O., Bryant D.A., Dismukes G.C. (2010). Boosting autofermentation rates and product yields with sodium stress cycling: application to production of renewable fuels by cyanobacteria. Appl. Environ. Microbiol..

[123] Guerra L.T., Xu Y., Bennette N., McNeely K., Bryant D.A., Dismukes G.C. (2013). Natural osmolytes are much less effective substrates than glycogen for catabolic energy production in the marine cyanobacterium *Synechococcus* sp. strain PCC 7002. J. Biotechnol..

[124] bitop AG Extremolytes. http://www.bitop.de/cms/website.php?id=/en/index/extremolyte.htm.

[125] Kovacevic V., Wesseler J. (2010). Cost-effectiveness analysis of algae energy production in the EU. Energy Policy.

[126] Du W., Liang F., Duan Y., Tan X., Lu X. (2013). Exploring the photosynthetic production capacity of sucrose by cyanobacteria. Metab. Eng..

[127] Reed R.H., Warr S.R.C., Kerby N.W., Stewart W.D.P. (1986). Osmotic shock-induced release of low molecular weight metabolites from free-living and immobilized cyanobacteria. Enzyme Microb. Technol..

[128] Fulda S., Hagemann M., Libbert E. (1990). Release of glucosylglycerol from the cyanobacterium *Synechocystis* spec. SAG 92.79 by hypoosmotic shock. Arch. Microbiol..

[129] Erdmann N., Zuther E., Abarzua S. (1992). Comparative studies on the photoproduction of nonhydrogenous resources by cyanobacteria. Curr. Microbiol..

[130] Takagi M., Karseno, Yoshida T. (2006). Effect of salt concentration on intracellular accumulation of lipids and triacylglyceride in marine microalgae *Dunaliella* cells. J. Biosci. Bioeng..

[131] Pal D., Khozin-Goldberg I., Cohen Z., Boussiba S. (2011). The effect of light, salinity, and nitrogen availability on lipid production by *Nannochloropsis* sp. Appl. Microbiol. Biotechnol..

[132] Waditee R., Bhuiyan M.N., Rai V., Aoki K., Tanaka Y., Hibino T., Suzuki S., Takano J., Jagendorf A.T., Takabe T., Takabe T. (2005). Genes for direct methylation of glycine provide high levels of glycinebetaine and abiotic-stress tolerance in *Synechococcus* and Arabidopsis. Proc. Natl. Acad. Sci. USA.

[133] Klähn S., Marquardt D.M., Rollwitz I., Hagemann M. (2009). Expression of the *ggpPS* gene for glucosylglycerol biosynthesis from *Azotobacter vinelandii* improves the salt tolerance of *Arabidopsis thaliana*. J. Exp. Bot..

[134] Sievers N., Muders K., Henneberg M., Klähn S., Effmert M., Junghans H., Hagemann M. (2013). Establishing glucosylglycerol synthesis in potato (Solanum tuberosum L. cv. Albatros) by expression of the ggpPS gene from Azotobacter vinelandii. J. Plant Sci. Mol. Breed..

[135] Kaczmarzyk D., Anfelt J., Särnegrim A., Hudson E.P. (2014). Overexpression of sigma factor SigB improves temperature and butanol tolerance of *Synechocystis* sp. PCC6803. J. Biotechnol..

[136] Takeda T., Miyao K., Tamoi M., Kanaboshi H., Miyasaka H., Shigeoka S. (2003). Molecular characterization of glutathione peroxidase-like protein in halotolerant *Chlamydomonas* sp. W80. Physiol. Plant..

[137] Yoshimura K., Miyao K., Gaber A., Takeda T., Kanaboshi H., Miyasaka H., Shigeoka S. (2004). Enhancement of stress tolerance in transgenic tobacco plants overexpressing *Chlamydomonas* glutathione peroxidase in chloroplasts or cytosol. Plant J..

